# Histopathological Analysis of Esophageal Damage Caused by Coin-Shaped Lithium Batteries in Living Piglets

**DOI:** 10.7759/cureus.71598

**Published:** 2024-10-16

**Authors:** Shinsuke Ohashi, Daisuke Kanamori, Sayuri Kaji, Goki Uchida, Tetsuro Sugihara, Kazuaki Miyaguni, Nei Fukasawa, Shoko Handa, Masashi Kurobe, Takao Ohki

**Affiliations:** 1 Pediatric Surgery, The Jikei University School of Medicine, Tokyo, JPN; 2 Pathology, The Jikei University School of Medicine, Tokyo, JPN; 3 Vascular Surgery, The Jikei University School of Medicine, Tokyo, JPN

**Keywords:** button battery injury, esophageal foreign body, esophageal injury, esophageal stenosis/chemically induced, pig

## Abstract

Background: More than 3,000 cases of accidental ingestion of coin-shaped lithium batteries (CSLBs) have been reported in the United States. Battery ingestion can cause serious injury and even death. Prior reports have indicated that complications often occur two or more hours after ingestion. However, to date, the temporal changes in esophageal damage remain unclear. To address this knowledge gap, we examined the histological features associated with these temporal changes.

Methods: Six piglets were used as models. After laparotomy and thoracotomy, three CSLBs were inserted into the esophagus of each pig. The esophagi were removed for histological examination at 0.5, 1, 2, 4, 6, and 8 hours. The consumed capacities of the batteries were measured after removal.

Results: Mucosal damage began at the margins of batteries, gradually spreading to the centers of the negative pole. At 0.5 hours after implantation, although necrosis at the limbus had reached the muscle layer, it became more extensive with time. At six hours, the full-thickness wall was damaged in all areas of the negative pole. The consumed capacity increased markedly after six hours, at which point holes opened in the outer case on the positive pole of the battery with observed electrolyte leakage. The consumed capacity was correlated with the amount of alkaline hydroxide ions.

Conclusion: Our study revealed changes over time in injury site and depth. Although early diagnosis and treatment are necessary, managing batteries to avoid complications is also important. Additionally, developing safer batteries is warranted.

## Introduction

With the spread of portable electronic and compact devices in recent years, the demand for small-sized 3 Volt (V) coin-shaped lithium batteries (CSLBs) and 1.5 V alkaline button batteries has increased rapidly. Regarding shape, coin-shaped batteries are 10-30 mm in diameter and 1.2-7.7 mm in thickness, while button batteries are approximately 4-12 mm in diameter and 1.2-6.0 mm in thickness. CSLBs have a high electromotive force (EMF) of approximately 3 V compared with alkaline button batteries (1.5 V). CSLBs are more dangerous due to their high EMF and shape, which makes them inherently more likely to become lodged in the esophagus. According to the National Capital Poison Center, there have been more than 3,000 cases of accidental ingestion of button batteries in the U.S. every year since 2004, with approximately 2,000 cases reported annually among children under six years of age. Although not limited to CSLBs, moderate or severe complications are reported in 2-3% of battery ingestion cases each year, while 1-5 deaths are reported annually [1]. In addition, many other cases of serious complications and deaths, including esophagotracheal fistulas, have been reported in relation to the accidental ingestion of CSLBs [2-6].

Regarding the devices associated with accidental ingestion of CSLBs in recent years, TV and garage remote controls, game consoles and toys, wristwatches, and small lights have all been more frequent sources [1]. Clinically, complications often occur within two hours after accidental ingestion [7,8]. In cases resulting in death, the time elapsed between accidental ingestion and mortality is often unknown. However, the cause of death is thought to be corrosion of the esophagus by alkali chemicals generated by the contact of CSLB with the esophagus, formation of an esophageal arterial fistula, and massive bleeding from this site [5,6,8].

Tanaka et al. previously examined the histological evaluation of esophageal damage after accidental ingestion of CSLBs in dogs, observing partial necrosis of the esophageal muscle layer [9]. However, this study did not clarify the relationship with the battery contact site, and histological changes in esophageal damage over time have not yet been reported. Therefore, to address this knowledge gap, we histologically examined the temporal changes in esophageal injury caused by CSLBs at battery contact sites using living piglets that were similar in size to the esophagus of one- to two-year-old human infants, who are particularly prone to accidental ingestion.

## Materials and methods

All experiments were conducted in compliance with the animal experiment regulations of The Jikei University School of Medicine and in accordance with the animal experiment protocol under approval number 2016-026.

One Landrace × Large White × Duroc (LWD) piglet weighing approximately 12 kg was used as a control examination, and six LWD piglets were used as the experimental models in this study. Regarding preoperative management, food intake was restricted until 09:00 p.m. on the day before the experiment, while drinking water intake was restricted until two hours before the experiment. Anesthesia was induced by intramuscular injection of medetomidine 0.06 mg/kg and midazolam 0.3 mg/kg; general anesthesia was maintained by continuous inhalation of isoflurane. The trunk was immobilized in the supine position, with the limbs restrained, and lactate Ringer’s solution was infused intravenously through the ear vein. The piglets were intubated and placed on a ventilator, wherein oxygen saturation and heart rate were monitored. We attempted inserting batteries orally several times as a preliminary experiment. However, due to individual differences in animal size, placing batteries in the same position in the esophagus was difficult. Therefore, we decided to insert batteries transgastrically.

We also decided to place three batteries in the esophagus of each animal to reduce the number of piglets to be euthanized from an animal welfare perspective.

**Figure 1:**
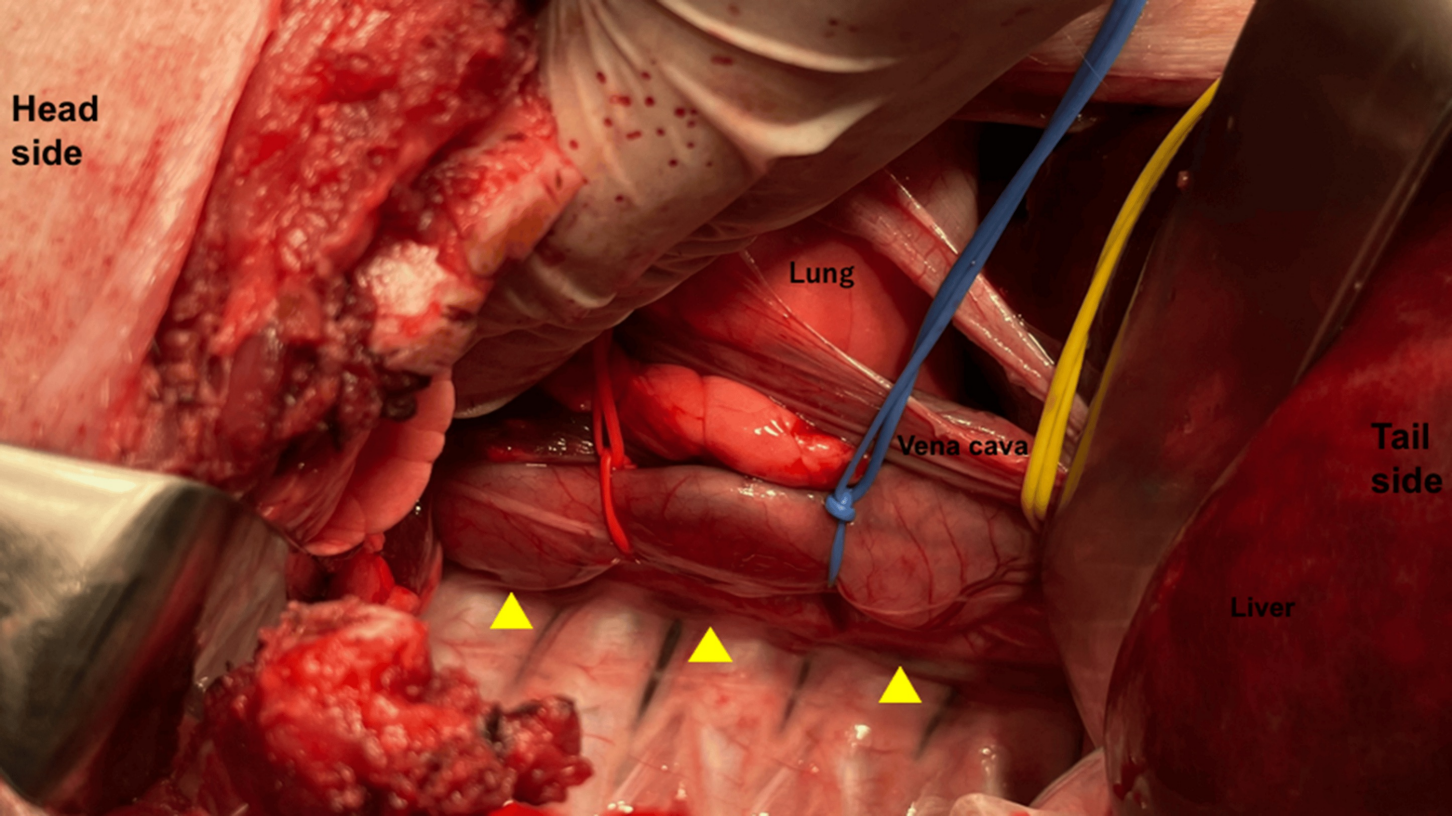
Overview of battery insertion. The battery is inserted transgastrically into the esophagus and loosely ligated with a vessel loop to prevent short-circuiting by contact. Yellow arrowheads indicate inserted CSLBs. CSLBs: coin-shaped lithium batteries

A median upper abdominal incision was made; the right side of the chest was opened; and the stomach and esophagus were identified after the dissection of the right inferior pulmonary ligament. The gastric body was incised; three commercially available unused CR2032 CSLBs (Panasonic, 20 mm in diameter, 3.2 mm thick, 3 V EMF) were manually inserted and implanted in the esophagus. The positive and negative pole surfaces of each battery were aligned, and the entire circumference of the esophagus was loosely ligated with a vessel loop® at the site between the batteries to prevent forming a short circuit due to contact between the batteries in the esophagus (Figure 1).

The esophagus was removed with the batteries retained in place at each of the pre-determined time points: 0.5, 1, 2, 4, 6, and 8 hours after the battery implantation. As a control for the experiment, about three, only the outer case, with the inner contents removed, was inserted into the esophagus and removed after six hours. The extracted esophagus was then opened along the long axis on the positive pole; the batteries were removed. The piglets were immediately euthanized by intravenous injection of potassium chloride solution after esophageal removal.

**Figure 2:**
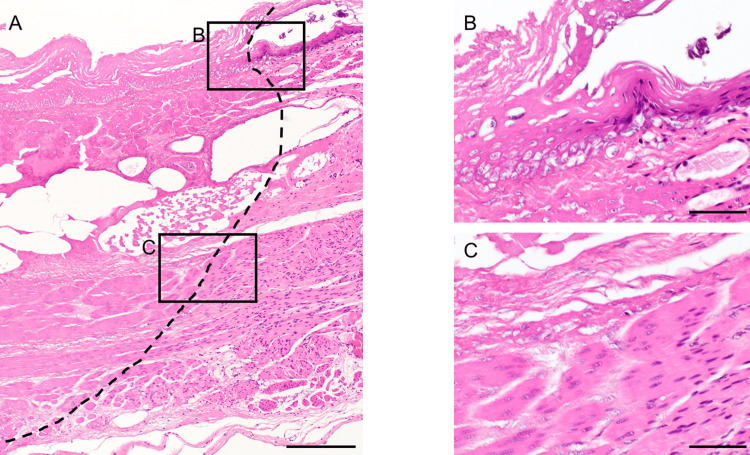
Definition of necrosis. A: The dashed line indicates the borderline of necrosis. B, C: High magnification images of each site. Necrosis was diagnosed based on whether the nuclei were stained or not. Scale bar represents 200 µm (A), 50 µm (B, C)

The extracted esophagi were fixed in formaldehyde (20%). Sections were prepared in the short-axis direction at the center of the battery and histologically observed by hematoxylin and eosin staining. Tissue damage was defined by the observation of cell necrosis. Specifically, necrosis was defined as poor staining of cell nuclei. They were diagnosed by a pathologist (Figure 2).

The degree of tissue necrosis at the battery edge (positive-negative boundary) and center of the negative pole was compared separately in the mucosal layer (from the epithelium to the submucosa), shallow muscle layer (inner ring muscle layer), deep muscle layer (outer longitudinal muscle layer), and adventitia. After the experiment, the battery was discharged at a constant flow rate of 1.5 mA until the battery voltage reached 1.5 V; the capacity (mAh) was measured. The consumed capacity (mAh) was calculated by subtracting the measured capacity (mAh) from the unused battery (nominal capacity: 220 mAh).

## Results

**Figure 3:**
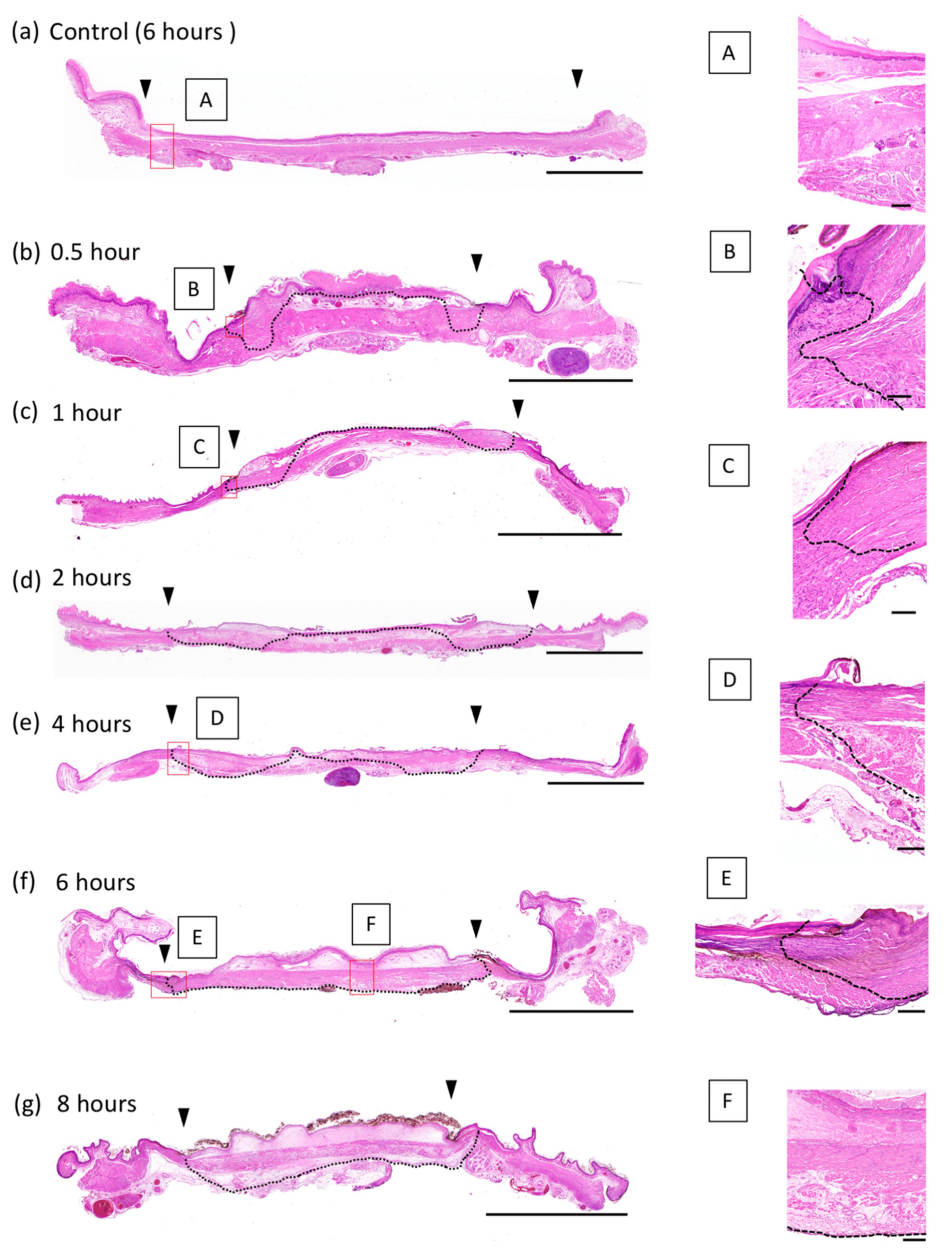
Histopathological findings (HE staining). A short-axis esophageal section is prepared at a site corresponding to the center of the battery. The boundary between the positive and negative poles is indicated by the arrowheads. The area between the black arrowheads corresponds to the negative pole. A-F are high-magnification images of the areas in the red boxes. Over time, the damage progressed both in-depth and over the entire negative pole surface. The necrotic area is indicated by a dashed line. Scale bar represents 100 µm (B, C), 200 µm (A, D, E, F), and 5 mm (a-g).

Esophageal damage initiated in the mucosal layer adjacent to the limbus near the negative pole and gradually spread to the center of the negative pole. At 0.5 hours after battery implantation, necrosis of the marginal area was already observed up to the muscular layer, with some extension to the deeper layers. However, the extent was small; the adventitia was generally preserved without total necrosis. Although the damage at the center of the negative pole remained in the mucosal epithelium of the mucosal layer until two hours later, necrosis extended deeper within the mucosal layer after four hours. After six hours, the findings of full-layer necrosis and adventitia damage were observed in all areas in contact with the negative pole (Figure 3).

On the other hand, the outer case, which was placed as a control, did not cause any damage at all.

**Figure 4:**
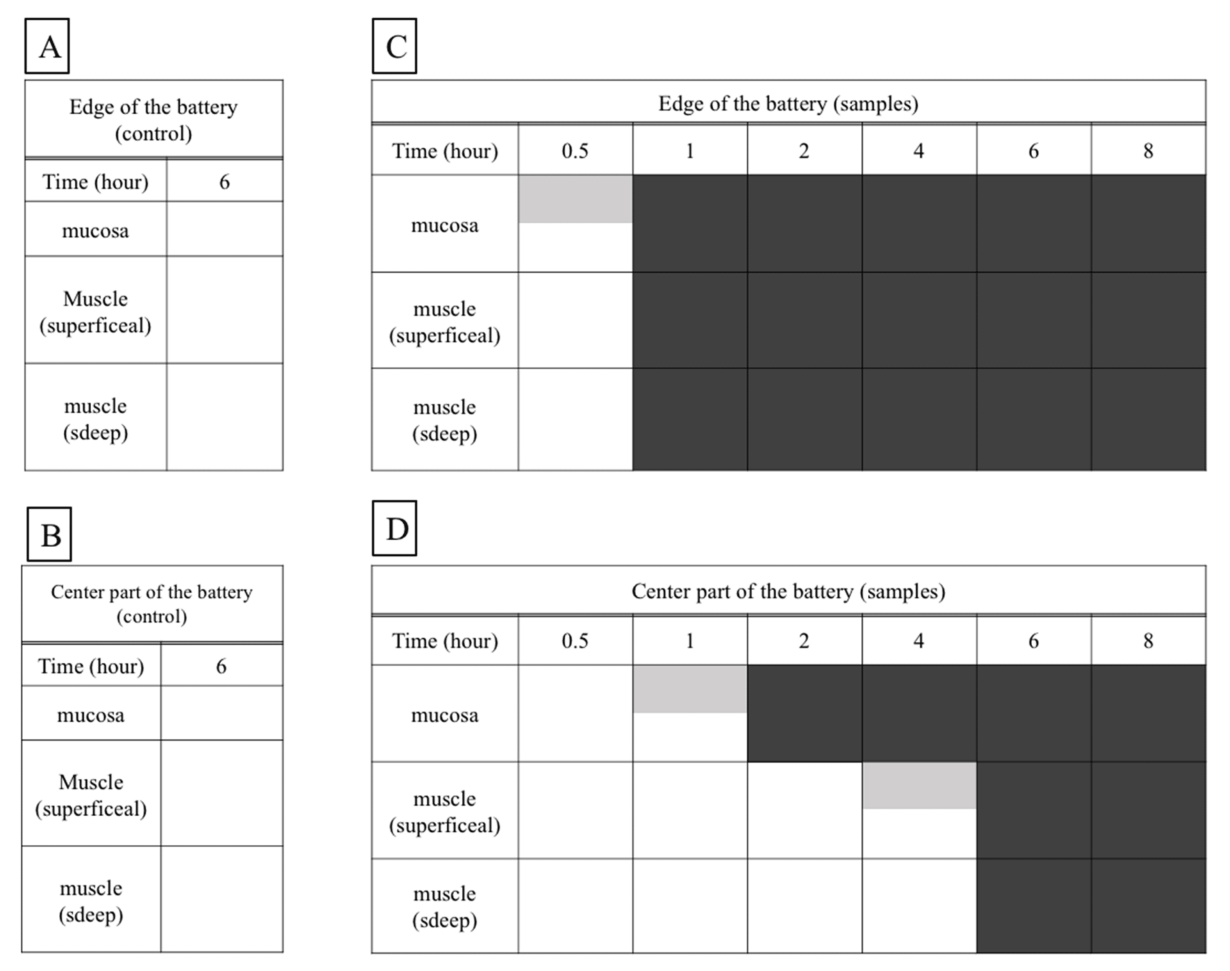
Schematic diagram of the esophageal damage over time. The edge area is damaged to the deep muscle layer in a short time, but the effect on the anode center is limited up to four hours. On the contrary, no necrosis occurred at all in the control. Gray boxes represent partial injury to each layer. Black boxes represent all of each layer being injured.

A schematic overview of this process is shown (Figure 4).

After two hours, neutrophil migration was observed in the border region between the necrotic and non-necrotic tissues. There was no evidence of tissue rupture suggesting perforation at any time point. No tissue damage was observed at the positive end after eight hours. Three specimens could be taken at each time; no difference in the degree of histological damage was observed in each. There were no instances of unexpected hemorrhage, damage to other organs, hypoxia, or death during the experiment.

**Figure 5:**
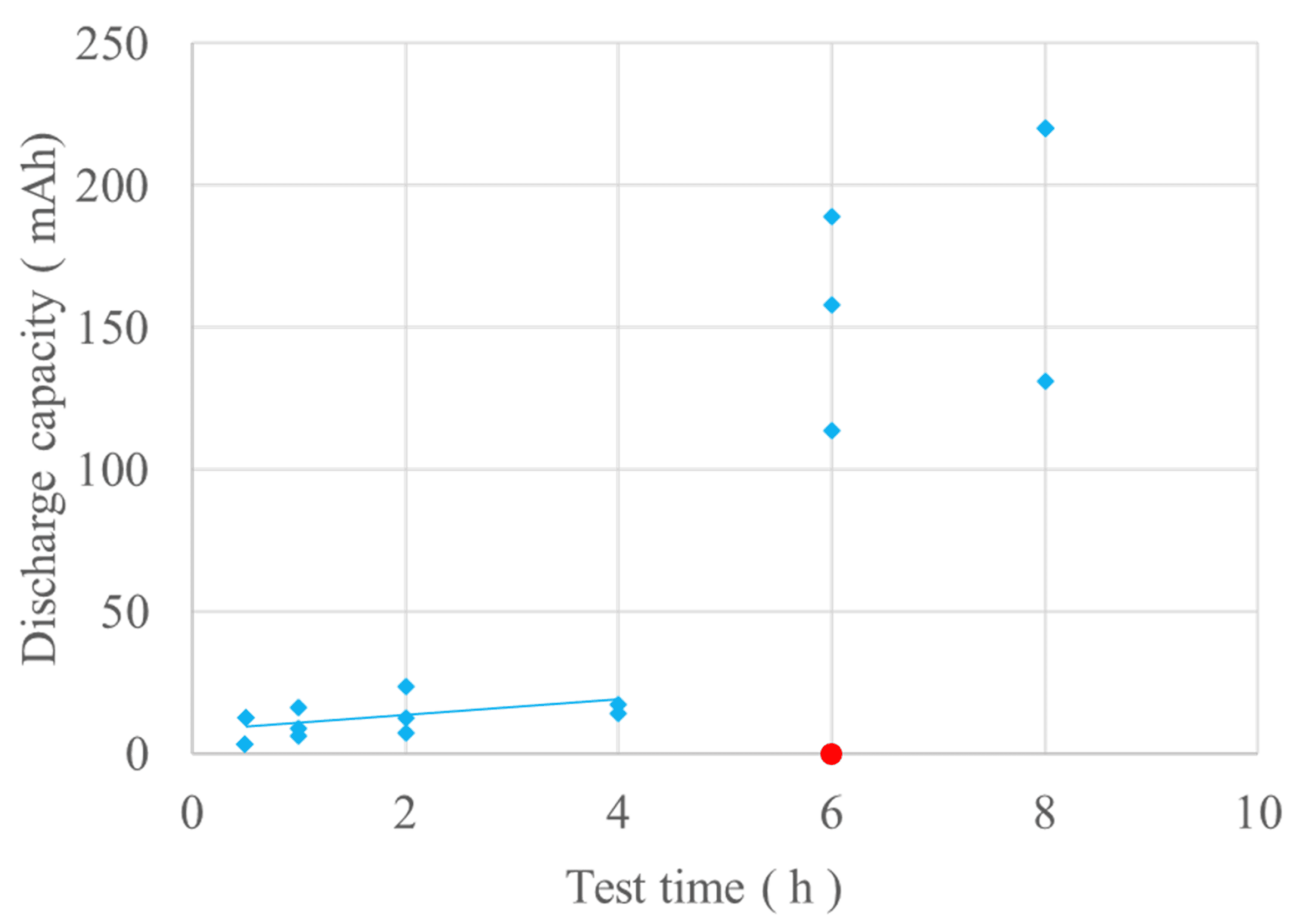
The results of the measurements of consumed capacity. The blue diamond-shaped plots show the consumed capacity of the excised batteries; there is a linear correlation up to four hours but a sharp increase after six hours. The red circle plot represents the control; consumed capacity was zero.

Measurements of the excised batteries showed that the consumed capacity due to discharge increased with the duration of detention, with a sharp increase observed after six hours (Figure 5).

**Figure 6:**
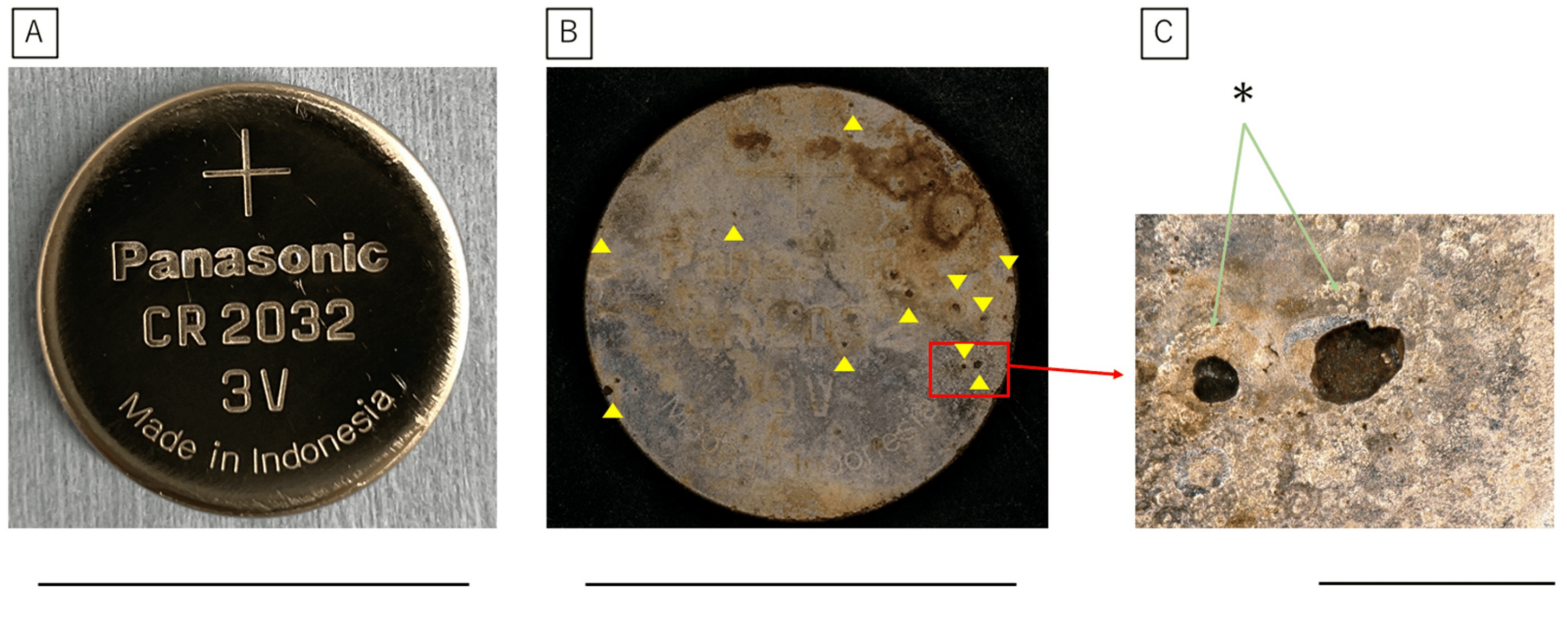
Appearance of the battery after removal. A: The positive pole surface of the control battery after four hours after insertion. B: The positive pole surface of the battery after six hours of insertion. Numerous small holes, shown as yellow arrowheads, are formed. C: Enlarged view of the red box. Leakage of the cathode material was observed (*). Scale bar represents 20 mm (A, B) and 1 mm (C)

Examination of the removed battery revealed small holes in the positive pole of the battery case after six hours of detention (Figure 6).

Leakage of the electrolyte from the inside of the battery was also observed.

## Discussion

**Figure 7:**
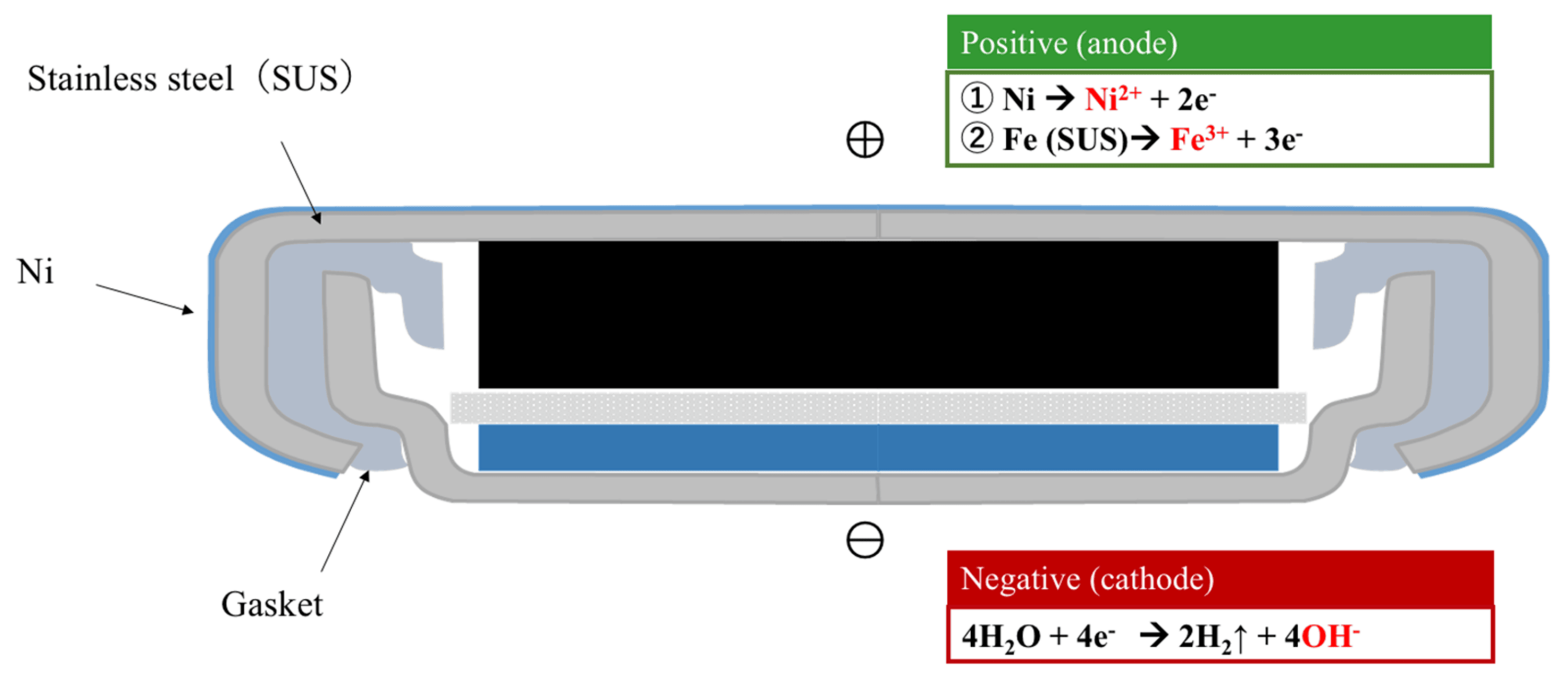
The structure of a coin-shaped lithium battery and chemical equations representing the chemical reactions that occur at the positive and negative pole. Image Credits: Shinsuke Ohashi with the cooperation of the Battery Association Japan.

The mechanism of esophageal tissue damage caused by accidental ingestion of a battery involves an electric current flowing from the positive electrode to the negative electrode through the esophageal wall, or the contact of saliva with the battery. At the positive pole, the tissue dissolves, and at the negative pole, water electrolysis occurs, resulting in the production of hydroxide ions (Figure 7).

Past studies using the esophagus of cadaveric pigs have shown that when CLBB was placed, the pH rose to pH 10.0 in 1.5 hours and to pH 12.0 after three hours [10]. This results in a strongly alkaline state, which is thought to cause tissue damage to the esophageal wall from the mucosal surface [8,10]. In our experiments, we found that necrosis of the esophageal tissue did not progress uniformly at the contact surface between the battery and the esophagus. However, necrosis started at the limbus of the CSLBs and progressed gradually to a wider and deeper layer. This mechanism is plausible because the limbus is the area where the anode and cathode are in close proximity and where most of the current flows.

Therefore, esophageal damage after accidental ingestion of CSLB is similar to that of corrosive esophagitis caused by accidental ingestion of an alkaline detergent. The course of esophageal damage is as follows: bacteria invade the tissue within 24-48 hours [11], after which fibroblasts proliferate in the wound, and collagen deposition occurs for one week. The wound then becomes fragile for up to three weeks owing to the shedding of necrotic tissue and the formation of new collagen. This repair process has also been observed in endoscopic submucosal dissection (ESD) of the esophagus, and various findings have been reported. The esophageal wall is histologically classified from the lumenal side into the mucosal layer, lamina propria, muscularis mucosa, submucosa, inner circular muscle layer, outer longitudinal muscle layer, and adventitia. Injuries beyond the mucosal lamina propria generally cause scarring during healing, which may result in strictures. Esophageal ESD involves the resection of the mucosal layer to the submucosa. There have been several reports that esophageal stricture occurs in more than 90% of cases of resection of more than 3/4 of the circumference [12], whereas other reports have indicated that only follow-up observation is sufficient for resections of less than 3/4 of the circumference [13]. Delayed perforation has been reported to occur from the day of ingestion to approximately three weeks after ESD. Conversely, fibrosis is thought to occur during the repair of necrosis in the muscle layer, while perforation occurs due to food retention or extension by balloon dilation. In one study of the effects of battery ingestion, including CSLBs, esophageal perforation was reported to occur within a few days to weeks after ingestion [14]. Therefore, if the damage exceeds more than 3/4 circumference beyond the submucosal layer, there is a possibility of stricture, while if it reaches the muscle layer, the risk of perforation should be considered.

The most common cause of death following CSLB ingestion is exsanguination due to a fistula formation between the esophagus and aorta. Akinkugbe et al. reported that erosions form within a few days to weeks after CSLB lodging, and a fistula with the aorta becomes apparent within a few weeks [15].

In our experiment, necrosis was observed even at 0.5 hours after ingestion of unused CSLB, with histological analysis confirming that tissue damage developed and progressed in a short time. In a previous clinical report, esophageal ulceration was observed two hours following accidental ingestion of CSLB [8]. However, in our experiment, full-thickness wall necrosis was observed in only a portion of the esophagus at one hour, suggesting that ulcer formation and perforation may occur even one hour after accidental ingestion of CSLBs. Necrosis of the entire anode surface up to the adventitia was completed between four and six hours following ingestion, which is generally consistent with a report of esophageal perforation that started five hours after ingestion [16]. Even after eight hours, the mucosa in contact with the positive pole surface of the battery was not damaged. Thus, no tissue damage was observed beyond 1/2 the circumference. Therefore, esophageal stricture is unlikely to occur as a complication of accidental ingestion. However, there have been many reports of stricture cases. This is not considered a simple comparison because, unlike ESD, it is related to the depth of the mucosa to be damaged and the fact that it takes time to extend slowly into the muscle layer. In addition, the progression of esophageal injury is likely to vary depending on the size of the esophagus, shape, EMF, and the condition of use of the ingested battery. In the present study, no tissue rupture was observed histologically, suggesting that perforation was unlikely to occur during the acute phase.

Our results showed that the consumed capacity of the battery increased with the length of time it remained in the esophagus, with a positive correlation observed up to four hours but significant variations after six hours. At six hours, small holes in the positive pole of the battery case were observed, which is thought to have caused leakage of the battery contents and significant battery deterioration due to the intrusion of body fluids and other substances into the battery interior. As described above, the consumed capacity of a battery is correlated to the amount of produced alkaline hydroxide ions. Regarding consumed capacities of less than 20 mAh (only 10% of the unused battery capacity) for up to four hours, full-thickness wall necrosis was limited to the battery edges. However, at the center of the negative pole, the extent of necrosis extended deeper beyond the mucosal layer to the muscle layer, but not up to the full-thickness wall. After six hours, necrosis was observed on the entire surface of the anode up to the full-thickness wall, suggesting that the leakage of battery contents, in addition to the formation of hydroxide ions due to energization, was largely responsible. A noteworthy finding of this experiment is that even a battery that appears to have been used could cause damage to the muscle layer of the esophagus if approximately 10% of its capacity remains. The management of used batteries is also important.

The goals that should be met to ensure the development of safer batteries are as follows: (1) ensure that there are no holes in the battery; (2) reduce the consumed capacity of the battery to limit the amount of produced hydroxide ions and thus limit the depth of esophageal necrosis. The ultimate goal of these modifications is to reduce the reactive capacity of the battery to zero. We look forward to the efforts of the battery manufacturers.

The most common treatment for CSLB ingestion is endoscopic removal of the battery, while measures to prevent subsequent complications are important. Conservative treatment is sometimes indicated in cases of perforation. However, subtotal esophagectomy has been reported to be necessary in many cases [17]. To avoid esophageal injuries caused by accidental ingestion of CSLBs, companies that manufacture batteries and products using batteries have recently implemented measures to make it difficult for infants to remove new batteries from packs and to remove batteries from products. Furthermore, new batteries are being developed to reduce the effects of accidental battery ingestion on the human body. Therefore, the results of this study should contribute to confirming the effectiveness of measures in preventing accidental CSLB ingestion. The most important consideration when limiting the damage caused by battery ingestion is to prevent accidental ingestion. Further, households and medical personnel should be aware of the seriousness of esophageal damage caused by CSLBs and educate themselves to prevent accidental ingestion.

Limitations

Individual differences among piglets, especially in esophageal thickness, could affect the results of the experiment. Although we did not measure the diameter of the esophagus in this experiment, the piglets were about the same thickness, but in rare cases, some of them appeared thinner. To evaluate this accurately, it is necessary to increase the number of animals, but this is difficult because they are large animals and it is undesirable for animal welfare.

The battery was inserted transgastrically and could be affected by gastric acid. A method of inserting the battery orally should be considered.

The piglet is under general anesthesia the entire time the battery is inserted and is in a different state than when the battery is actually placed. In other words, the effects of swallowing saliva and the vomiting reflex are not added. In the future, a model will be considered in which artificial saliva is injected after battery insertion.

In this experiment, the piglets were euthanized after removal of the esophageal specimen. Of clinical importance are the histologic changes in the esophagus after battery removal. In the future, a protocol is needed to allow piglets to survive after battery removal and observe changes over time.

## Conclusions

It is well known that esophageal damage occurs early after the accidental ingestion of CSLBs. In this study, we have clarified the histological changes in esophageal damage over time. This is also the first report to clarify that there is a correlation between the consumed capacity of the battery and histological damage. The results revealed that even when the remaining battery capacity was 10%, damage could occur in the full-thickness wall of the esophagus.

This experiment reaffirmed that removing accidentally ingested batteries as soon as possible is very important. Moreover, it is also important for the battery industry to educate the public by advising against accidental ingestion and improving batteries that are less likely to discharge even if accidentally ingested.
